# Reintroducing genetic diversity in populations from cryopreserved material: the case of Abondance, a French local dairy cattle breed

**DOI:** 10.1186/s12711-023-00801-6

**Published:** 2023-04-19

**Authors:** Alicia Jacques, Grégoire Leroy, Xavier Rognon, Etienne Verrier, Michèle Tixier-Boichard, Gwendal Restoux

**Affiliations:** 1 INRAE, AgroParisTech, GABI, Université Paris-Saclay, 78350 Jouy-en-Josas, France; 2 Food and Agriculture Organization, viale delle Terme de Caracalla, 00153 Rome, Italy

## Abstract

**Background:**

Genetic diversity is a necessary condition for populations to evolve under natural adaptation, artificial selection, or both. However, genetic diversity is often threatened, in particular in domestic animal populations where artificial selection, genetic drift and inbreeding are strong. In this context, cryopreserved genetic resources are a promising option to reintroduce lost variants and to limit inbreeding. However, while the use of ancient genetic resources is more common in plant breeding, it is less documented in animals due to a longer generation interval, making it difficult to fill the gap in performance due to continuous selection. This study investigates one of the only concrete cases available in animals, for which cryopreserved semen from a bull born in 1977 in a lost lineage was introduced into the breeding scheme of a French local dairy cattle breed, the Abondance breed, more than 20 years later.

**Results:**

We found that this re-introduced bull was genetically distinct with respect to the current population and thus allowed part of the genetic diversity lost over time to be restored. The expected negative gap in milk production due to continuous selection was absorbed in a few years by preferential mating with elite cows. Moreover, the re-use of this bull more than two decades later did not increase the level of inbreeding, and even tended to reduce it by avoiding mating with relatives. Finally, the reintroduction of a bull from a lost lineage in the breeding scheme allowed for improved performance for reproductive abilities, a trait that was less subject to selection in the past.

**Conclusions:**

The use of cryopreserved material is an efficient way to manage the genetic diversity of an animal population, by mitigating the effects of both inbreeding and strong selection. However, attention should be paid to mating of animals to limit the disadvantages associated with incorporating original genetic material, notably a discrepancy in the breeding values for selected traits or an increase in inbreeding. Therefore, careful characterization of the genetic resources available in cryobanks could help to ensure the sustainable management of populations, in particular local or small populations. These results could also be transferred to the conservation of wild threatened populations.

**Supplementary Information:**

The online version contains supplementary material available at 10.1186/s12711-023-00801-6.

## Background

Genetic variability in populations is necessary to allow for adaptation to changing environments. Indeed, highly diverse populations are more likely to have advantageous or adaptive allelic variations, which lead to a significantly higher evolutionary potential [1]. In addition, genetic diversity is essential for selection since it is directly linked to genetic variance. In dairy cattle, this selection is based on the use of a limited number of sires, resulting in both genetic drift and increased inbreeding throughout the genome [2]. Thus, the selection process inevitably leads to a reduction in genetic diversity, the intensity of which depends on selection intensity and breeding goals [3, 4]. Depending on the breed and its breeding scheme, the implementation of genomic selection could also impact genetic gain as well as genetic diversity, thus suggesting the need for careful monitoring in order to ensure the sustainability of these programs [5–7]. Indeed, it is necessary to monitor the level of genetic diversity of local breeds to take action based on their risk status [4, 8].

Cryopreservation and the development of gene banks are useful contributions to the conservation of genetic diversity in domestic animals. However, the use of ex situ genetic resources should be combined with in situ breed conservation, as mentioned in the FAO Global Plan of Action [9]. In France, the National Gene Bank was created in 1999 for the conservation of semen and embryos of domestic animal breeds with the aim of hosting samples that are representative of the genetic diversity of all French breeds. In the following years, the genetic collections grew rapidly, with a major contribution of the bovine species [10]. One of the main purposes of cryopreservation is the conservation of genetic diversity of threatened populations as the last resort; for instance one indicator of the Sustainable Development Goals of the FAO (SDG 2.5.1b) considers the number of breeds with a sufficient amount of stored genetic material to reconstitute them in case of extinction [11]. However, cryopreserved material can also be used before the extinction of a breed to reintroduce genetic diversity in order to limit inbreeding in populations [12]. However, the effective use of these resources for that purpose has remained quite rare, although it has already been demonstrated in a concrete case [13].

The long-term cryoconservation of reproductive material (semen, ova, or embryos) makes it possible to use ancient individuals in order to re-introduce lost genetic diversity in current farm populations [14]. However, the use of ancient genetic resources can hinder genetic progress for traits that are currently under selection [15]. The more a population has been subjected to strong selection, resulting in large genetic gain over successive generations, the more the conserved genetic resource will exhibit a lag in performance for the selected traits. While it is more common to use external genetic resources in current plant breeding programs, it is almost never the case in animal populations due to longer generation intervals that reduce the efficiency of bridging or pre-breeding strategies to fill the gap in performance [16, 17]. However, a theoretical study showed that the use of old Dutch cryobank bulls could increase the genetic variability and genetic merit in a current population of Holstein Friesian dairy cattle [18]. Another risk of re-using ancient sires for reproduction is the increase in inbreeding level of the population through mating with relatives in the current population. Thus, a real compromise has to be found between a positive contribution to genetic diversity, on the one hand, and the slowdown of genetic progress and possible increased inbreeding, on the other hand. Simulation studies have already shown the potential of using cryopreserved genetic material to bring back diversity [14, 15] but very few real cases have been reported until now [19]. A recent case illustrated the successful recovery of a lost lineage of the Y chromosome in the Holstein breed thanks to the use of frozen semen from the National Gene Bank in the USA [20].

In this study, we propose to analyze a concrete case where an ancient cryopreserved bull was used to restore genetic diversity in a selected French local dairy cattle breed, the Abondance breed. We used pedigrees, genotypes and genomic estimated breeding values to determine the impact of the re-use of this bull on the current Abondance breeding scheme with respect to both diversity and performance. The objective was to demonstrate the effectiveness of using ex situ genetic resources to reintroduce genetic diversity and to understand the key parameters that are related to the successful use of cryopreserved collections.

## Methods

### Description of the Abondance breed and animals

The Abondance breed originates from the Chablais region, which is the northernmost part of the French Alps, located between the French shoreline of Lake Geneva and the Valais canton in Switzerland. The geographical isolation of this area has favored the development of an original and hardy cattle population that is adapted to the mountain environment and to farming systems involving transhumance (see Additional file 1: Fig. S1). About 90% of its milk is processed into cheese, mainly cheeses that benefit from a Protected Designation of Origin (PDO). The Abondance breed is the fourth French dairy cattle breed in population size, with about 48,000 cows, but it represents only 1.4% of the total French dairy herd. Selection through progeny testing has been conducted for decades and thus, pedigrees and performances are available for many individuals. Genomic selection has been applied since 2015, giving access to genomic estimated breeding values (GEBV) that are comparable between animals of different cohorts. Finally, cryopreserved material is available in collections in breeding companies and at the French National cryobank.

Crossbreeding of Abondance cows with Red Holstein bulls took place during the 1980s in order to improve both milk yield and udder morphology. Then, some crossbred artificial insemination (AI) bulls (75% Abondance, 25% Red Holstein) were proposed for sale by the end of the 1980s to mate cows on farms. Starting from this time, some original purebred families, such as the Amiens bull lineage, no longer produced approved sires. Finally, this strategy was discontinued at the end of the 1990s and was accompanied by a forced return to a pure breed that may have decreased the genetic variability of the breed due to a bottleneck effect. This trend motivated the breeders’ association to reintroduce genetic diversity by using cryopreserved material in the early 2000s. The Abondance breed is therefore a good case study to investigate the consequences of reintroducing genetic diversity from a former breeding stock.

In the current study, we focused on the use of cryopreserved semen from Naif, a bull born in 1977 and a son of Amiens. Its semen was used during two distinct periods, first between 1980 and 1993 and then between 2004 and 2009. We defined two cohorts of contemporary genotyped sires. Cohort 1 corresponds to 62 sires that produced offspring born between 1980 and 1993 along with Naif. Cohort 2 corresponds to 165 sires that produced offspring born between 2004 and 2009, corresponding to the period when Naif was re-used. The Cohort 1 sires were born between 1970 and 1991 and the Cohort 2 sires were born between 1982 and 2007. Then, we defined four female cohorts corresponding to the dams that produced progeny during the same two periods and for which two indices, the dairy merit index and the milk production index, were available. Cohort 1a corresponds to 2443 dams that were mated to sires other than Naif and produced offspring between 1980 and 1993. Cohort 1b consists of the 37 females mated to Naif during the same period. Three females were mated with Naif as well as with another sire for the period 1980–1993 and were thus present in both cohorts. Cohort 2a corresponds to 4092 dams that were mated to sires other than Naif and produced offspring between 2004 and 2009. Cohort 2b corresponds to 25 dams that were mated to Naif during the same period. Fifteen females were present in both cohorts, as they were mated with Naif and other sires during the period 2004–2009. The distribution of the birth years of the four female cohorts are available in Additional file 2: Fig. S2. Finally, we also defined the 2017 cohort as the current population consisting of all individuals born in 2017, in order to study the long-term effect of re-using Naif.

### Pedigree data

We used a dataset that included all ancestors of the available genotyped individuals in 2017 in the Abondance breeding scheme extracted from the national database. Thus, the pedigree included 25,010 individuals born from 1944 to 2018. The quality of the pedigree was evaluated through the equivalent number of generations with the NGEN module of the PEDIG software [21]. Pedigree quality was computed as the average between males and females of the equivalent number of generations for the two production periods of Naif (1980–1993 and 2004–2009), the two periods corresponding to the birth years of sires from Cohort 1 (1970–1991) and Cohort 2 (1982–2007), as well as for the 2017 cohort.

The genetic contribution of Naif to the gene pool of a given cohort was defined as the probability that, at any neutral locus, an allele drawn at random in the genotype of a randomly chosen animal in this cohort originates from Naif. This probability was computed from pedigree data using the PEDIG software. The total contributions of Naif were calculated for each year from 1980 to 2017. Then, two types of contributions were defined, an old contribution from the first use of Naif (1980–1993) and a recent contribution from the contemporary use of the cryopreserved semen from Naif (2004–2009). These contributions were calculated using the same method as for the total contributions. The contemporary contribution of Naif was computed by giving it a new identifier when it was used in the second period. Thus, we can compute distinct contributions of Naif when it was used during either the first or the second period since it is considered as two different individuals in the pedigree.

### Molecular data

We used the genotypes of 6958 individuals obtained with the 50K single nucleotide polymorphism (SNP) chip (Illumina Infinium® BovineSNP50 BeadChip). Quality control was performed by removing SNPs with a call rate lower than 99% and individuals with less than 99% genotyped SNPs. Once this quality control was done, no other SNPs were deleted and 43,801 autosomal SNPs remained in the data sets. However, 26 pairs of SNPs were found to have identical positions in the genome and were removed from the analyses. On average, the marker density was one SNP every 57.2 ± 60.0 kb.

#### Measurements of heterozygosity

The individual observed heterozygosity of Naif, and of sires of the two cohorts, were computed and the mean heterozygosity of Cohorts 1 and 2 was compared using a two-way ANOVA.

#### Measurement of inbreeding

Inbreeding was assessed from molecular data using runs of homozygosity (ROH). ROH represent long autozygous segments of the genome (i.e. identical-by-descent). Here, a ROH was defined as a homozygous segment of at least 15 SNPs and 1000 kb long, with at least one SNP every 70 kb. Two consecutive SNPs could not be included in the same ROH if they were separated by more than 140 kb. ROH were detected using the “homozyg” PLINK 1.9 function [22, 23]. The size of the sliding window was set to 15 SNPs. The number of heterozygous calls in the sliding window was limited to 1, and the upper limit for missing data was 5. Inbreeding estimates based on ROH, were calculated according to McQuillan et al. [24], as the proportion of the genome included in ROH as follows:

$$FROH_{i} = \frac{{\Sigma LROH_{i} }}{Lgen},$$

with $\Sigma LROH_{i}$ is the total length (in bp) of ROH for individual $i$ and $Lgen$ is the length (in bp) between the first and the last SNP covering the part of the genome considered.

The 1148 genotyped individuals born in 2017 were grouped according to their relatedness to Naif, forming two groups: one group with a recent link to Naif, which appears to be a father on the pedigree during its second use, and the other group with no link to the recent use of Naif.

#### Genetic structure by multivariate analysis

Principal component analyses (PCA) were conducted with Cohorts 1 and 2 using the ade4 package [25]. Naif was added as a supplementary individual to the PCA (i.e. it does not contribute to the construction of the principal components).

Then, a between-class analysis (BCA) was carried out on the 2017 cohort to specify the family links of the individuals with Naif. The aim of this analysis was to maximize the between-group variance while minimizing the within-group variance. The 1148 individuals were separated into six classes representing the possible combinations of the different uses of Naif: either the absence of any link, or the presence of one or two old or recent links (i.e. due to its first or second use through either a single parent or both of them). Eighty-five individuals had no family link with Naif (0_LWN), 49 individuals had a recent link with Naif through one of their parents (1_LWN_R), 387 individuals had an old link with Naif through one of their parents (1_LWN_O), 479 individuals were related to the old use of Naif through its both parents (2_LWN_O), one individual was related to the recent use of Naif through its both parents (2_LWN_R), and 147 individuals had one old and one recent link with Naif (2_LWN_OR) (see Additional file 3: Fig. S3).

### Genomic performance data evaluated in 2017

Genomic estimated breeding values, GEBV, were assessed in 2017 for all genotyped individuals. We extracted these values for Cohort 1, Cohort 2 and Naif from the French national genomic evaluation facility database. Since genomic performance data were missing for some bulls from Cohort 1, the GEBV calculated for Cohort 1 correspond to the 54 bulls evaluated out of the 62 available. We also extracted these values for the 15 sons and 25 grandsons of Naif from its second use, as well as for the 155 bulls in Cohort 2 with no genetic link to the second use of Naif, their 416 sons and 528 grandsons for which GEBV were available.

For the male cohorts, we focused on three multi-trait indices: (i) the total merit index (ISU, *Indice de Synthèse Unique*), (ii) the dairy merit index (INEL, *Indice National Economique Laitier*) and (iii) the reproduction merit index (REPRO). For the female cohorts, we focused on two milk indices, the milk yield (MILK) and the dairy merit index (INEL). All these values were centered around the 2017 mobile base (i.e. mean values of the dams for the period 2009–2011) and scaled according the 2017 mobile base estimated genetic variances. The mean was set to 100 for ISU and to 0 for REPRO and INEL. For both ISU and INEL, one genetic standard deviation (sd) unit corresponds to 20 points while for REPRO one point corresponds to one genetic sd unit.

### Statistical analyses

All statistical analyses and graphical representations were performed using R [26] and the ggplot2 package [27]. Statistical tests were performed using the lm function, post-hoc tests were conducted using the emmeans package [28], and type II ANOVA were performed using the car package [29].

## Results

### Pedigree data

#### Quality of the genealogy

The pedigree quality for the two production periods of Naif ranged from 3.19 (sd = 0.41) equivalent number of known generations for the 1980–1993 cohort, to 5.80 (sd = 0.40) for the 2004–2009 cohort. The equivalent number of known generations was 7.57 (sd = 0.08) for the 2017 cohort. The equivalent number of known generations for Cohort 1 (1970–1991) and Cohort 2 bulls (1982–2007) were 2.96 (sd = 0.73) and 4.12 (sd = 0.92), respectively. As expected, the quality of the genealogies increased over time, explaining the different values between the two cohorts (see Additional file 4: Fig. S4).

#### Progeny of Naif

**Fig. 1:**
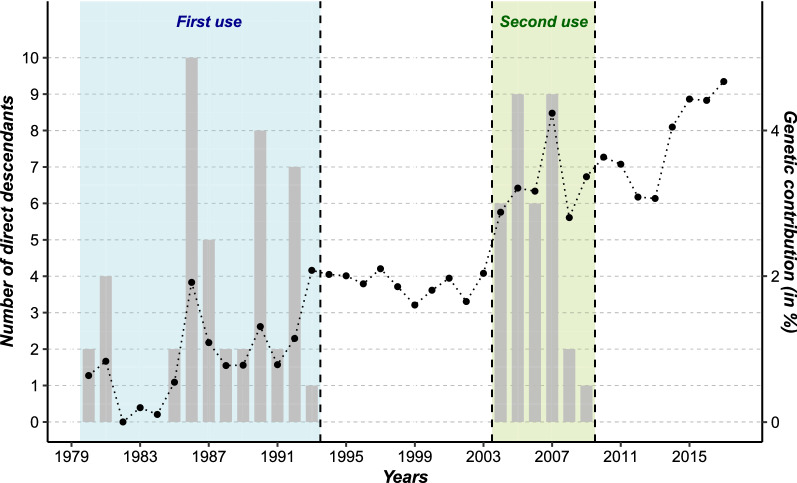
Production of Naif’s direct progeny and total annual pedigree-based genetic contribution from 1980 to 2017. Blue: first period of the use of Naif; and green: second period of the use of Naif

During its first use, Naif produced 45 direct offsprings born between 1980 and 1993 (Fig. 1). In the second period, its semen was used to produce 33 offsprings born from 2004 to 2009. Thus, Naif produced 3.2 offsprings per year during its first period of use and 5.5 offsprings per year when its semen was re-used. Naif was no longer used for artificial insemination after 2009.

#### Genetic contribution of Naif

**Fig. 2:**
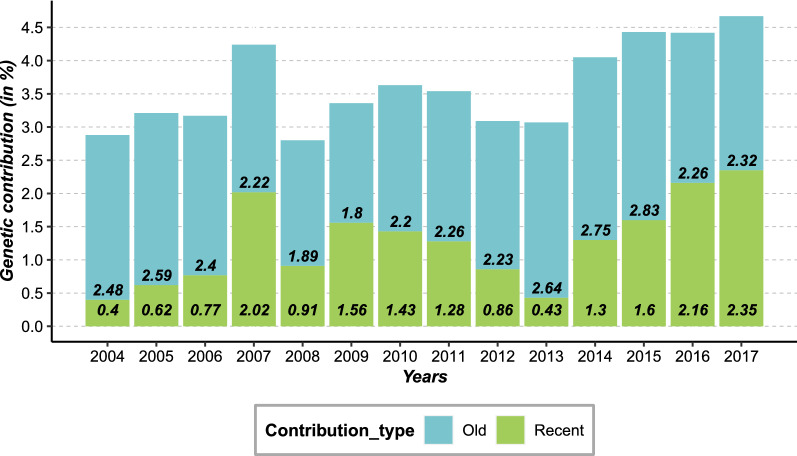
Old and recent contribution of Naif evaluated from pedigree data from 2004 to 2017. In blue: old contribution from the first period of the use of Naif; and in green: recent contribution from the second period of the use of Naif

During the first period of use, the overall genetic contribution of Naif to the population increased from 1980 to 1993 because of the increasing number of offspring over time. Then, from 1994 to 2003, its genetic contribution remained fairly constant due to the use of its offspring at a similar level as that of the offspring of its contemporary sires (i.e. average sire) although it no longer produced direct offspring. During its second period of use, from 2004 to 2009, the contribution of Naif increased again because of the increased number of its direct offprings. From 2009 onwards, a slight decline appeared during the following four years, followed by a marked increase from 2014 to 2017 due to the intense use of Naif’s progeny (Fig. 1). Distinguishing between past and recent contributions of Naif (Fig. 2) revealed the impact of each period of the use of Naif: older contributions had a larger impact than recent ones, with the exception of the year 2017 for which the recent contribution surpassed the old contribution. The recent contribution globally increased over time, in particular starting in 2013.

### Molecular data

#### Measurements of heterozygosity

**Fig. 3:**
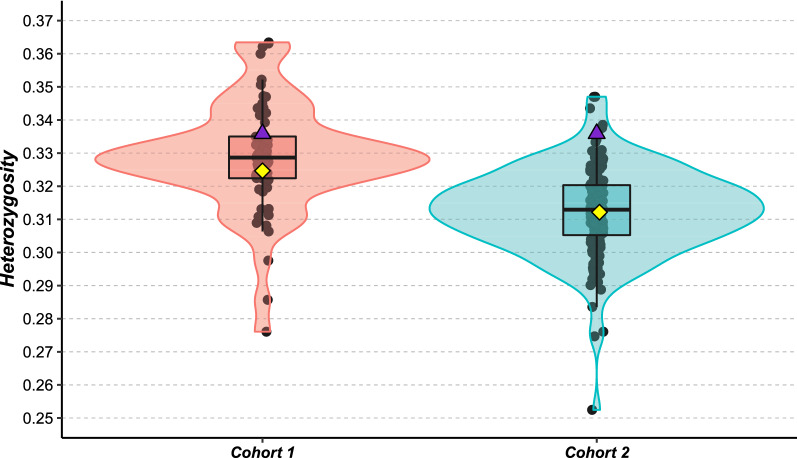
Average heterozygosity of contemporary cohorts for both uses of the Naif bull. The 62 bulls in Cohort 1 are shown in pink, the 165 bulls in Cohort 2 are shown in blue, Naif is represented by the purple triangle, the mean of each cohort corresponds to the yellow square

The average heterozygosity of the 62 sires from Cohort 1 was 32.9% (sd = 1.6) while that of the 165 sires from Cohort 2 was 31.3% (sd = 1.3). Across the whole genome, the average heterozygosity decreased significantly between the first and the second cohort (ANOVA, F = 60.5, df = 1, p < 0.05). Naif had an individual heterozygosity rate of 33.6%, which corresponds to the third quartile of the distribution of Cohort 1, while it was one of the most heterozygous individuals of Cohort 2 (Fig. 3).

#### Inbreeding measurements

**Fig. 4:**
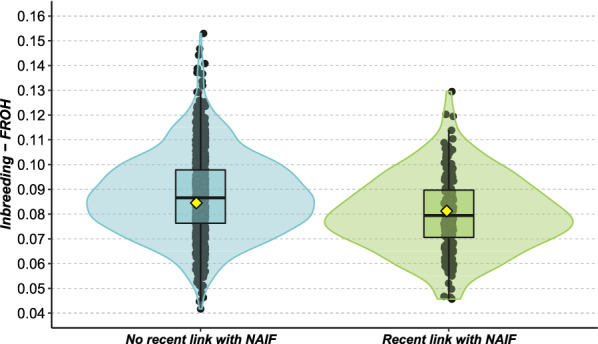
Inbreeding of individuals of cohort 2017 depending on their link with the recent use of Naif. Blue: individuals that do not originate from the recent use of Naif; and green: individuals that originate from the recent use of Naif

For the 2017 cohort, 85 animals were genetically unrelated to Naif (0_LWN), 436 animals had a single family link with Naif through either the maternal or paternal side (1_LWN) and 627 animals had two family links with Naif through both parents (2_LWN), with average inbreeding of 8.67% (sd = 1.81), 8.64% (sd = 1.58) and 8.58% (sd = 1.62), respectively. Inbreeding did not differ significantly according to the number of links with Naif (ANOVA, F = 0.274, df = 2, p = 0.76). In addition, 197 individuals had a link to Naif from its second period of use while 951 individuals were not related to the recent use of Naif. Mean inbreeding was 8.74% (sd = 1.62) and 8.01% (sd = 1.51) for unrelated or related individuals, respectively, with the recent use of Naif (Fig. 4). This difference was significant (ANOVA, F = 34.06, df = 1, p < 0.05).

#### Genetic structure by multivariate analysis

**Fig. 5:**
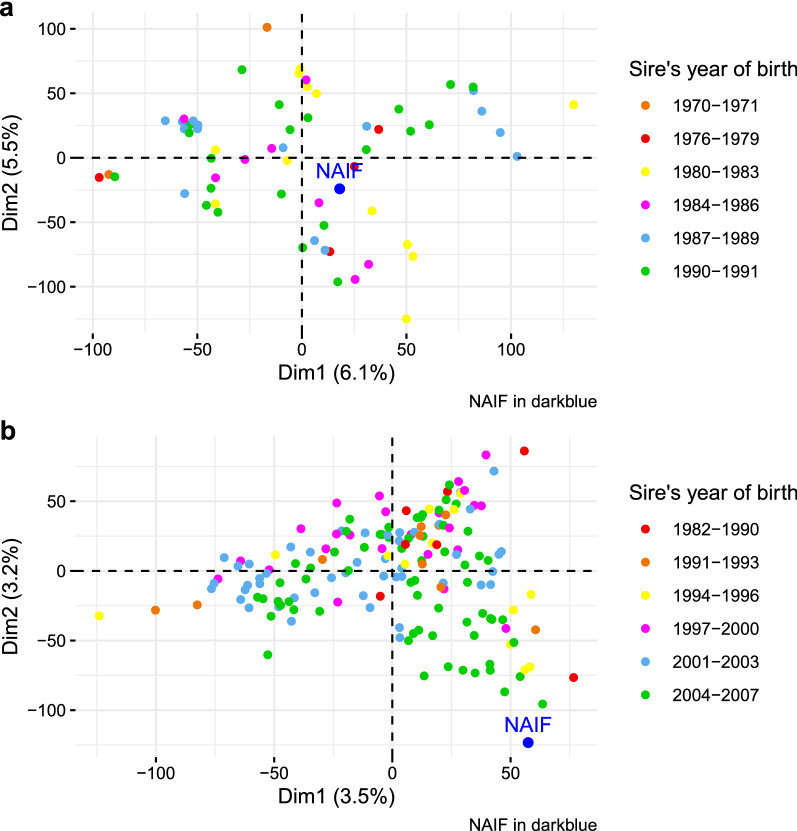
Principal component analysis of genotyping data for Cohort 1 (**a**) and Cohort 2 (**b**). Naif is represented by the blue dot

The first two components of the PCA of Cohort 1 explain 11.6% of the total inertia. Naif appears to be an “average” individual within Cohort 1 as it is positioned close to the origin of these two components (Fig. 5a). In the PCA related to its second use, Naif appears to be more distinct from the “average” sires of Cohort 2, with a more extreme position with respect to the first two components (Fig. 5b). The two first axes accounted for 6.7% of the total variability. In addition, individuals with a link to Naif (LWN, n = 85), i.e. for which Naif is in their pedigrees, appear to be well-separated from individuals with no link with Naif (no_LWN, n = 80) on this PCA (see Additional file 5: Fig. S5).

**Fig. 6:**
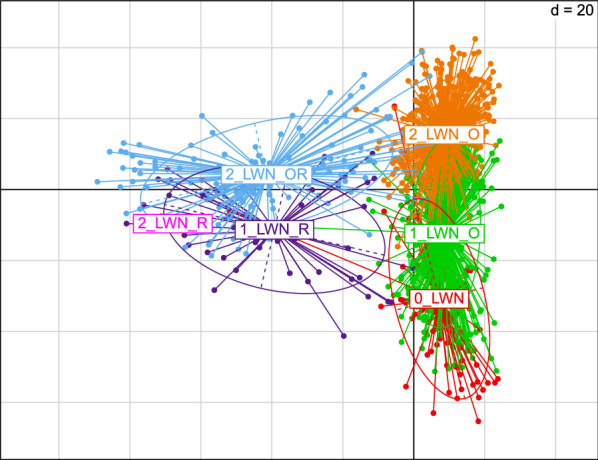
Between-class analysis of genotyping data of cohort 2017. Red: individuals not related to Naif; green: individuals with one old link with Naif; orange: individuals with two old links with Naif; blue: individuals with one old and one recent link with Naif; purple: individuals with one recent link with Naif; and magenta: individual with two recent links with Naif

For the BCA of the 2017 cohort, the three groups corresponding to a recent use of Naif (1_LWN_R, 2_LWN_R, 2_LWN_OR) appear to be well-separated from the other three groups with no link with Naif (0_LWN) or with links due only to its first use (1_LWN_O, 2_LWN_O) (Fig. 6).

### Breeding values

#### Males

**Fig. 7:**
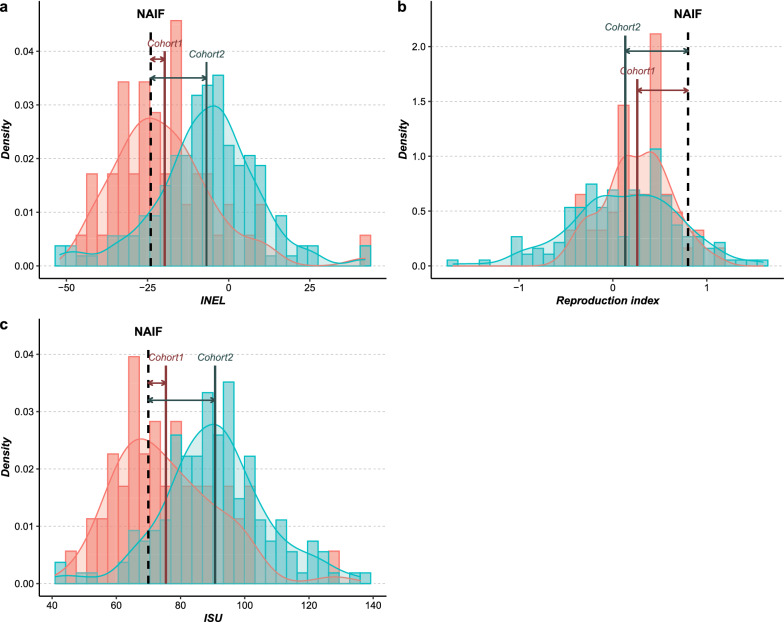
Distribution of INEL (**a**), reproduction index (**b**) and ISU (**c**) for Cohort 1 and Cohort 2. The 62 bulls in Cohort 1 are shown in pink, the 165 bulls in Cohort 2 are shown in blue, Naif is represented by the black dashed line, the mean of each cohort is represented by the solid line and the different gaps between Naif and the mean of each cohort by the arrows

The ISU, INEL and REPRO values of Naif were 70, -24 and 0.8, respectively, those for Cohort 1 were 75.46 (sd = 15.72), − 19.70 (sd = 15.85) and 0.26 (sd = 0.36) and for Cohort 2 were 90.75 (sd = 16.29), − 6.84 (sd = 15.86) and 0.13 (sd = 0.57), respectively (Fig. 7). The mean ISU and INEL for Cohorts 1 and 2 were significantly different (ANOVA, F = 36.42, df = 1, p < 0.05 for ISU; F = 26.78, df = 1, p < 0.05 for INEL). ISU differences of 5.46 and of 20.75 were found between Naif and Cohort 1 and between Naif and Cohort 2, respectively, and INEL differences of 4.30 and 17.16 were found between Naif and Cohort 1 and between Naif and Cohort 2, respectively. There was no significant difference between the means of the REPRO index for the two cohorts (ANOVA, F = 2.457, df = 1, p = 0.12).

**Fig. 8:**
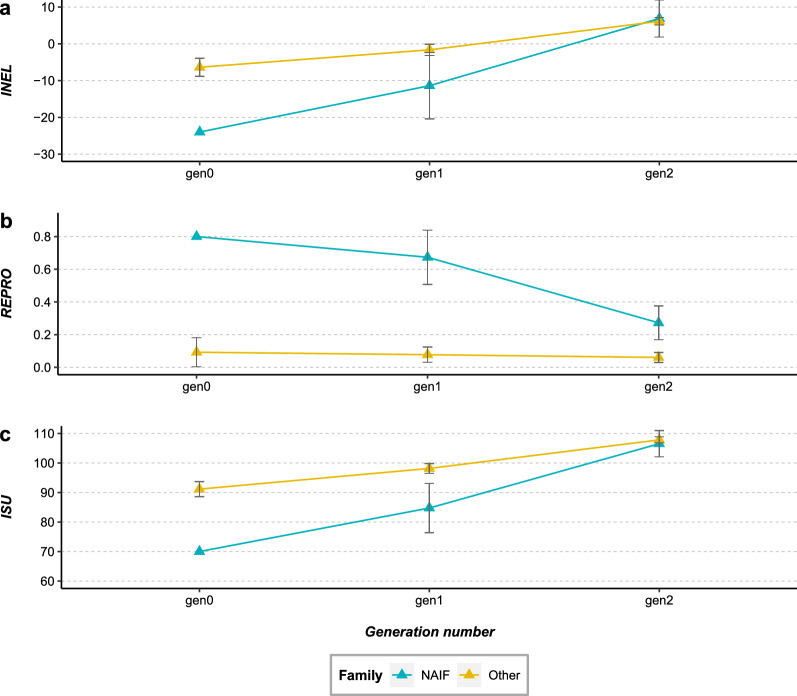
Average values of INEL (**a**), reproduction index (**b**) and ISU (**c**) in the two generations following the second use of Naif. The average values of the offspring from the reuse of Naif’s frozen semen are represented in blue and those of the offspring from the other sire families are represented in yellow. Error bars correspond to confidence intervals

The ISU, INEL and REPRO values for the 15 sons and 25 grandsons of Naif, from its second use, as well as the values of the 155 bulls of Cohort 2 without a genetic link to Naif’s second use and of their 416 sons and 528 grandsons, are shown in Fig. 8 (see Additional file 6: Table S1). For the three indices at generation 0, the ISU and INEL mean values for Naif were significantly lower than those for the other bulls, but the REPRO value was significantly higher (one sample t-test, t = 16.33, df = 154, p < 0.05 for ISU; t = 14.05, df = 154, p < 0.05 for INEL; and t = − 15.72, df = 154, p < 0.05 for REPRO). At generation 1, the ISU and INEL mean values for the Naif lineage were significantly lower than those for the other bull lineages, but the REPRO value was significantly higher (ANOVA, F = 8.93, df = 1, p < 0.05 for ISU; F = 5.46, df = 1, p < 0.05 for INEL; F = 22.42, df = 1, p < 0.05 for REPRO). At generation 2, the ISU and INEL mean values were no longer significantly different between the two lineages (ANOVA, F = 0.28, df = 1, p = 0.60 for ISU; F = 0.10, df = 1, p = 0.76 for INEL), but the REPRO value for the Naif lineage was still significantly higher (ANOVA, F = 8.61, df = 1, p < 0.05). Moreover, we computed the empirical within-family genetic variances of bull GEBV for the three indices within each sire family present in the pedigree at generation 1. The ISU, INEL and REPRO variances between the male progeny of Naif (i.e. half-sib family) were 271.64, 319.11 and 0.11, respectively, while the average values for the other sire male half-sib families were 194.80, 190.72 and 0.21, respectively.

#### Females

**Fig. 9:**
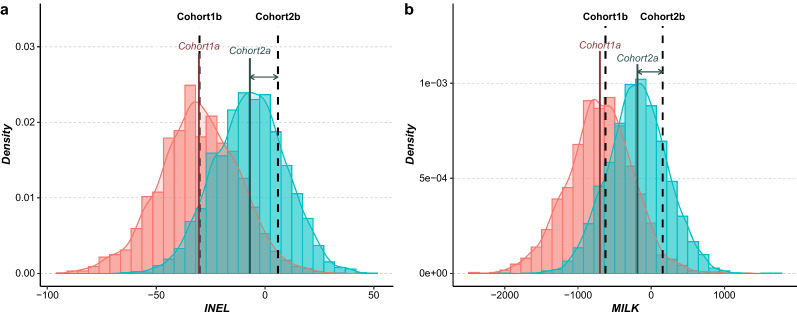
Distribution of INEL (**a**) and a milk index (**b**) for females mated with Cohort 1 and Cohort 2. The 2443 cows in Cohort 1a are shown in pink, the 4092 cows in Cohort 2a are shown in blue, the mean of each cohort is represented by the solid line, the performance mean of the Cohort 1b and Cohort 2b are represented by the black dashed lines and the gaps between the different cohort means by the arrows

The distribution of the INEL and MILK values for Cohort 1a and Cohort 2a and the relative position of the average values for Cohort 1b and Cohort 2b are shown on Fig. 9 (see Additional file 7: Table S2). The difference between the MILK and INEL means of Cohorts 1a and 1b were − 74.97 and − 0.25, respectively, and those between the means of Cohorts 2a and 2b were − 345.77 and − 12.90, respectively. For the INEL and MILK indices, the means between Cohort 1a and Cohort 1b were not significantly different but those of Cohort 2a and Cohort 2b were significantly different (ANOVA, F = 978.2, df = 3, p < 0.05 for INEL; and F = 768.6, df = 3, p < 0.05 for MILK).

## Discussion

The bull called Naif (born in 1977) used for AI belonged to a lineage that has since been lost, namely the progeny of a bull called Amiens. Naif was chosen to help “purify” the breed, i.e. to help decrease the proportion of the Red Holstein breed within the Abondance breed. This bull had the advantage of having a large stock of cryopreserved semen available. Thus, it was a good candidate to successfully reintroduce genetic diversity, in particular with a genetic origin from the Amiens lineage. Naif procreated 45 offspring in its first use and, after a 10-year period of inactivity, its semen was used once again to produce 33 offspring in a second use over a shorter time period, indicating a more intensive use of its semen. The years from 2004 to 2007 were marked by an important production of individuals with Naif as a sire, with 30 descendants in those four years alone. These two successive uses led to a large genetic contribution of Naif to the population. Its overall contribution increased in both periods of use and stagnated in between the periods. However, its contribution continued to increase after 2009, even after Naif was no longer used as a sire, through the use of its progeny in the Abondance breeding scheme. Moreover, while the original contribution remained nearly stable from 2004 to 2017, recent contributions increased from 2014 onwards and became almost equivalent to the original contribution. This growing trend shows that the offspring of Naif were not excluded from the Abondance breeding scheme, and in fact appear to have been increasingly used. Although their ISU values were initially lower than the contemporary average, we note that the use of progeny of Naif was primarily motivated by the reintroduction of an ancient lineage in the breed and the improvement of reproductive traits.

The analysis of performances revealed that Naif was an average individual during its first use. However, a discrepancy was observed when compared to a more recent cohort, due to the effect of on-going selection for production traits (see Additional file 8: Fig. S6). Indeed, at the time of its second use, Naif had much lower values for total merit index, ISU, and dairy merit index, INEL, than its contemporary sires (Fig. 8). A gap of nearly two generations between Cohorts 1 and 2 resulted in an increase of 15.29 (0.76 genetic sd unit). and 12.86 (0.64 genetic sd unit) points for the ISU and INEL, respectively. Such a lag in performance in a selected breed was expected and has already been highlighted in previous studies [14, 15]. This difference is likely to be even greater under more intensive selection resulting in larger genetic gain, as is the case for mainstream dairy cattle breeds such as the Holstein breed. Thus, one might expect that the larger the annual genetic gain, the more detrimental the effect of older individuals on the performance for selected traits. Leroy et al. [15] showed by simulations that an effective successful integration of cryopreserved sires with lower genetic values into breeding schemes would only be possible in the case of a marked change of breeding objectives that would give an advantage to their progeny. In the case of Naif, we found that this gap in performance can be effectively absorbed in a few generations through appropriate mating plans with elite females that could compensate for this lag. Indeed, we showed that females mated with Naif during its second use exhibited better breeding values than females mated with other contemporary bulls, although all these females were born in the same period. Moreover, within two generations, the difference in breeding values had already begun to decrease. Naif’s indices were 21.1 (1.05 genetic sd units) and 17.6 (0.88 genetic sd unit) points lower for ISU and INEL, respectively, compared to contemporary sires during its second use. After two generations, when comparing Naif’s lineage versus all other lineages, this gap was almost filled with a difference of only 0.07 genetic sd unit for ISU in favor of other lineages. Similarly, for INEL the gap almost disappeared with a difference of only 0.04 genetic sd unit but in favor of Naif’s lineage. A latency period is still necessary in order to fill this gap in performance, with a duration that is proportional to its magnitude. In plant breeding, Allier et al. [16] showed that collaborative diversity panels (i.e. genetic resources and elite lines) coupled with genomic prediction seemed relevant to enrich elite germplasm in maize. However, they found that “bridging” and “pre-breeding” steps favor the insertion of genes from genetic resources by limiting the mismatch on the traits of interest for breeding [17]. Similarly here, the qualities brought back by Naif were integrated into the breeding scheme mainly through its progeny. This was revealed by the continuously increasing genetic contribution of Naif to the Abondance breed after the end of its second use, and in particular its increasing contribution from the year 2013. Thus, as suggested by Eynard et al. [14] who used simulations, we demonstrated based on a long-term on-farm experiment that adding older bulls to a current breeding population undergoing selection can efficiently reintroduce genetic variability without having too great an impact on genetic merit. However, we emphasize that the case of NAIF is somewhat particular, as the primary goal was the reintroduction of a lost lineage. In a broader perspective, it could be relevant to use the optimal contribution strategy (OCS) to choose the appropriate candidate(s) in cryobanks in order to reintroduce genetic diversity without overly affecting genetic gain [30]. Furthermore, OCS could also be used to remove non desirable alleles from a population, such as the contribution of the Red Holstein breed in our case [31, 32].

Another important point is that the high level of heterozygosity of Naif could give access to a large gametic variance (i.e. a large genetic variability of its gametes). This high gametic variance, which was associated with a large number of offspring during its reuse, resulted in a rather large panel of diversity among its progeny, which could then be exploited in the breeding scheme. Indeed, the empirical genetic variances for the three indices (INEL, REPRO and ISU) of the sons of Naif after its reuse were all larger than the corresponding average within-family genetic variances of other contemporary bulls. Other studies have also revealed that the use of parents that produce more variable gametes may provide a better response to selection by increasing the probability of reproducing high-level genotypes [33, 34]. Thus, this criterion should be taken into account when choosing sires from cryobanks that are to be used in a contemporary breeding scheme.

However, in spite of the negative impact on performance traits that were under constant selection, the use of Naif increased the performance of other traits, in particular those that were not or only weakly selected. In our study, the use of Naif contributed performances for reproduction traits, which were not necessarily as much improved as other production traits included in the past breeding goals (reproduction traits were not or poorly evaluated before the 2000s). Indeed, while Naif was 0.7 genetic sd unit ahead of the other sires used at the time of its second use for REPRO, after two generations, its progeny was still 0.2 genetic sd unit ahead. The gain obtained for a production index such as INEL, but also the progress retained on the REPRO index, allowed the delay due to Naif’s progeny on the ISU total merit index to be absorbed. This underlines the fact that traits such as fertility, calving ease or vitality at birth, which are strongly affected in many dairy breeds by selection that focuses on production traits [35–37], could be improved in the future by using cryopreserved resources. This confirms that a change in breeding goals is one reason why old genetic resources may become more valuable in breeding schemes, as highlighted by Leroy et al. [15] based on simulations.

The study of the 2017 population, nearly two generations after the second use of Naif, highlighted a successful reintroduction of diversity within the breeding scheme. Inbreeding did not increase, and in fact a slight decrease in inbreeding was observed between individuals resulting from recent Naif matings and the rest of the population. This significant difference in inbreeding can be explained by the choice of the females that were mated with Naif. Indeed, bringing back an old bull can favor inbreeding if matings are conducted with related individuals (i.e. its descendants). This can be even stronger if the cryo-preserved individual has been heavily used in the past. Conversely, in the case of Naif we observed a decrease in inbreeding of its offspring resulting from its second use (see Additional file 9: Fig. S7). This case was rather favorable since the Amiens family, to which Naif belongs, had almost disappeared from the breeding scheme, or at least was no longer used to produce sires. More generally, the longer the time lapse between first and second use of sires, the lower the kinship, and thus the lower is the impact on the inbreeding of the population. In addition, Doekes et al. [38] showed that recent inbreeding is more detrimental than that due to more distant generations, since it leads to stronger inbreeding depression in many traits due to a shorter action of selection against inbreeding load (i.e. purging).

Multivariate analyses revealed the genetic originality of Naif. When first used, Naif was representative of its contemporary sires. However, when used for the second time, Naif appeared to be a more genetically original individual compared to the other active sires. The BCA computed two generations after the last use of Naif revealed that all individuals with a recent link to Naif can be distinguished from other individuals, thus highlighting the conservation of Naif’s genetic originality in its descendants. While the initial motivation was to return to a “pure” breed, the use of a cryopreserved individual was also able to reintroduce part of the genetic specificity of a lost family into a current population, thus increasing genetic variability. This neutral variability is potentially beneficial for adaptation to new conditions but also for the selection of individuals towards new breeding goals in the future.

Finally, in addition to the beneficial consequences in terms of neutral diversity and improved performances for new traits, the introduction of old genetic material can also have a negative impact, particularly on genetic load and its consequence, inbreeding depression. Indeed, the reintroduction of external or old genetic resources increases the risk of reintroducing deleterious alleles that have been absent or eliminated in the target population, which draws a parallel with the field of conservation biology where genetic rescue is currently used in endangered wildlife populations [39, 40]. This method aims at reducing inbreeding and restoring genetic diversity in small populations. However, in some cases, when the migrant individuals originate from large populations, they are likely to have a higher inbreeding load (i.e. recessive deleterious alleles segregating in the population often at low frequency but sometimes fixed [41]). Then, if the receiving population is small, the purging of these introduced deleterious alleles could be too costly (i.e. lower fitness and loss of diversity) thus increasing the risk of extinction [42]. This effect depends on the balance between the loss of diversity, the initial decrease in fitness and the reduction of genetic load [43]. In domestic animal populations, many genetic defects are regularly detected, and breeding programs tend to eliminate them from the population (e.g. Scrapie disease in sheep [44], and Bovine Leukocyte Adhesion Deficiency in bovines [45]). In these cases, the reintroduction of such diseases from old material can be detrimental. In the current study, we did not note any increase in inbreeding depression in the progeny of the reintroduction programs compared to the rest of the population (see Additional file 10: Fig. S8). However, we should emphasize the importance to cope with inbreeding depression by considering both the inbreeding level and the genetic load in breeding or conservation programs.

## Conclusions

This study is one of the first to document a real case of the long-term impact of the use of cryopreserved reproductive material in a domestic animal breeding scheme, both in terms of performances and genetic diversity. We showed that in spite of a lag in production traits, the use of appropriate mating plans can fill this gap within a few generations. Furthermore, these disadvantages can be balanced by gains for other traits for which breeding goals have changed since the conservation of genetic resources, for instance in the case of functional traits. In terms of genetic diversity, the use of cryopreserved material from ancient individuals can be beneficial by providing genetic originality to the current population that may otherwise be lost over time. In addition, the effectiveness of using old germplasm for genetic improvement also depends on the gametic variance of the reintroduced material, which should be as large as possible. At the very least, this gametic variance should be checked through the heterozygosity or, when feasible, through the haplotype diversity of donors. However, particular attention should be paid when re-using a sire that was massively used in the past, since it can increase the inbreeding level of the population by mating with its own descendants. Thus, the inbreeding rate should be managed by using planned matings with non- or weakly-related individuals. Nevertheless, the use of extinct lineages or very original individuals should have a positive impact on genetic diversity. In this respect, molecular data obtained from routine genotyping can be used to characterize the level of genetic diversity that could be contributed to the current population by sires from a gene bank. Indeed, genomic selection allows for the evaluation of former individuals on the same scale as that of current populations, even for traits that were not selected in the past. Considering that cryopreserved genetic resources are seldom used in breeding programs today, our work provides some indications to explain how successful the use of cryopreserved material can be. Further studies are needed to tailor recommendations for using cryopreserved resources to different objectives and expectations for each species and breed (e.g. management of a genetic defect, improvement of disease resistance, reintroduction of diversity, etc.). Finally, our approach could easily be transposed to wild populations for which the reintroduction of genetic diversity from zoos or reserves is a major concern.

## Supplementary Information

**Additional file 1: Figure S1.** Cows from the Abondance breed ruminating on high altitude pastures in the Chablais mountains, © Étienne Verrier (August 2022).

**Additional file 2: Figure S2.** Distribution of birth years for the four female cohorts. The 2443 cows in Cohort 1a are represented in pink, the 4092 cows in Cohort 2a are represented in blue, the 37 cows in Cohort 1b are represented in green and the 25 cows in Cohort 2b are represented in purple.

**Additional file 3: Figure S3.** Classification of different genetical links with the Naif bull for the 2017 cohort. Red: individuals with no genetic family link to Naif (0_LWN); purple: individuals with a recent genetic link with Naif through one of their parents (1_LWN_R); green: individuals with an old link with Naif through one of their parents (1_LWN_O); orange: individuals related to the first use of Naif by both parents (2_LWN_O); magenta: individuals related to the recent use of Naif by both parents (2_LWN_R); blue: individuals with one old and one recent genetic link with Naif (2_LWN_OR).

**Additional file 4: Figure S4.** Annual evolution of the equivalent number of generations from the pedigree data.

**Additional file 5: Figure S5.** Principal component analysis of genotyping data for Cohort 2. Green: individuals with no genetic link to Naif (noLWN); and red: individuals with a genetic link to Naif (LWN). Naif is represented by the blue dot.

**Additional file 6: Table S1.** Average values for the three indices (ISU, INEL, REPRO) at two generations after the second use of Naif.

**Additional file 7: Table S2.** Average values for the two indices (INEL and MILK) for female cohorts.

**Additional file 8: Figure S6.** Evolution of GEBV for the three indices (INEL, ISU, REPRO) in the Abondance breed. GEBV were assessed in 2017 for all genotyped individuals. Error bars correspond to standard errors.

**Additional file 9: Figure S7.** Annual evolution of average inbreeding (a) and average kinship (b) according to the re-use of Naif frozen semen. Inbreeding and relatedness values were evaluated from pedigree using the MEUW and PAR3 modules, respectively, of the PEDIG software. (a) Individuals resulting from the recent use of Naif are represented in green and individuals not resulting from the recent use of Naif are represented in blue. (b) Kinship within sires is shown in blue, kinship within dams is shown in red, and kinship between sires and dams is in purple. The kinships between Naif and other sires are represented in green. The kinships between Naif and dams are represented in orange. Error bars correspond to standard errors.

**Additional file 10: Figure S8.** Inbreeding depression on reproduction index in 2017 for individuals resulting or not from the reintroduction of the Naif bull. Blue: individuals not resulting from the recent use of Naif—Green: individuals resulting from the recent use of Naif. Regression lines were estimated using an ANCOVA model considering reproduction index (REPRO) as the response variable, and the inbreeding level based on ROH (FROH), the link to the recent use of Naif (NoRECENT_LWN if no link and RECENT_LWN if resulting for reintroduction of Naif) and its interaction as explanatory variables. In blue, $REPRO_{noRECENT\_LWN} = 0.25 - 1.99F_{ROH}$. In green, $REPRO_{noRECENT\_LWN} = 0.29 - 1.36F_{ROH}$. The slopes of the regression lines were not significantly different between the two groups (p = 0.63).
