# Supplementary figures and images for: Reintroducing genetic diversity in populations from cryopreserved material: the case of Abondance, a French local dairy cattle breed
Source: Genet Sel Evol. 2023 Apr 19;55:28. doi: 10.1186/s12711-023-00801-6 (PMC10114384; doi:10.1186/s12711-023-00801-6)

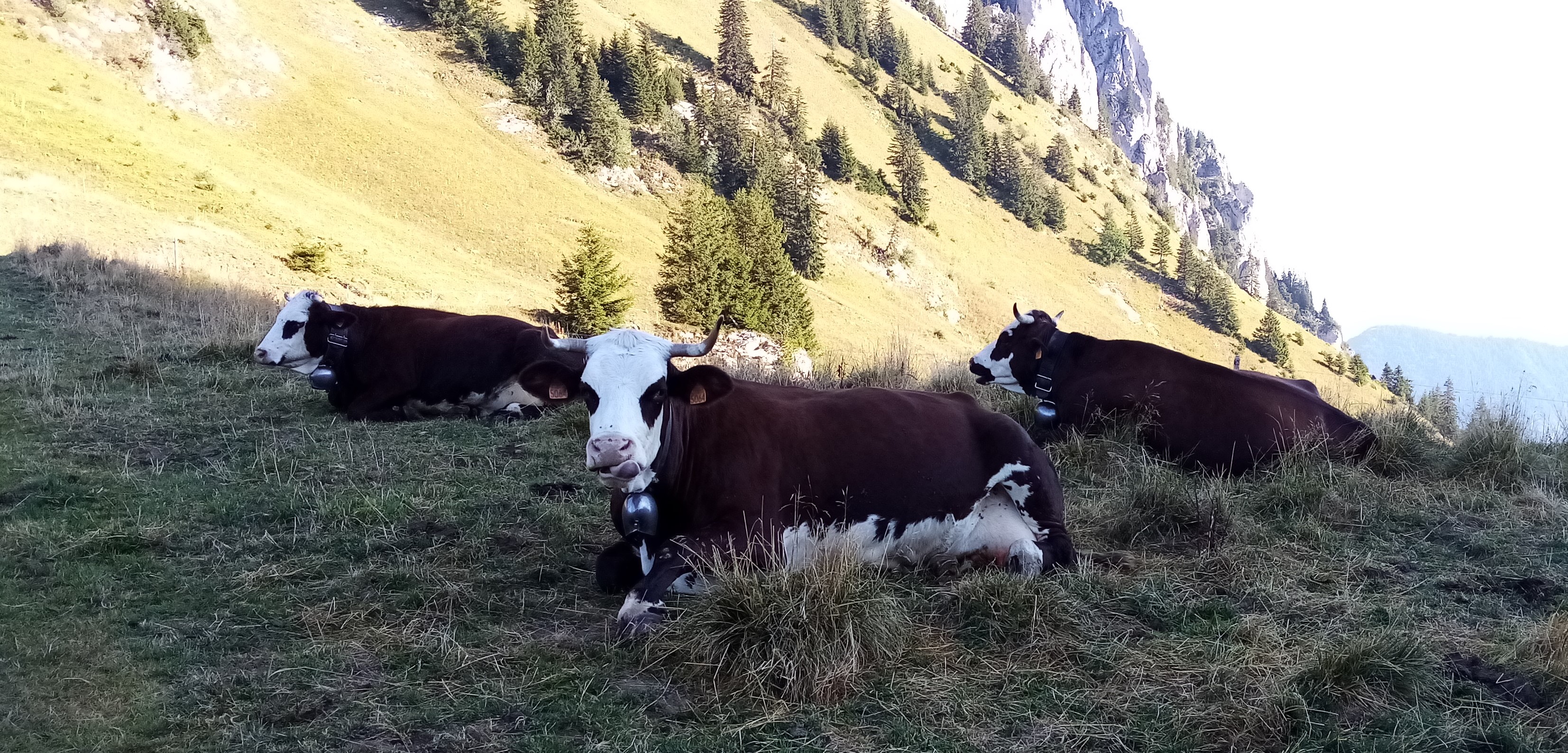

Supplement: Supplementary file 1 — Additional file 1: Figure S1. Cows from the Abondance breed ruminating on high altitude pastures in the Chablais mountains, © Étienne Verrier (August 2022). [file 12711_2023_801_MOESM1_ESM.jpg]

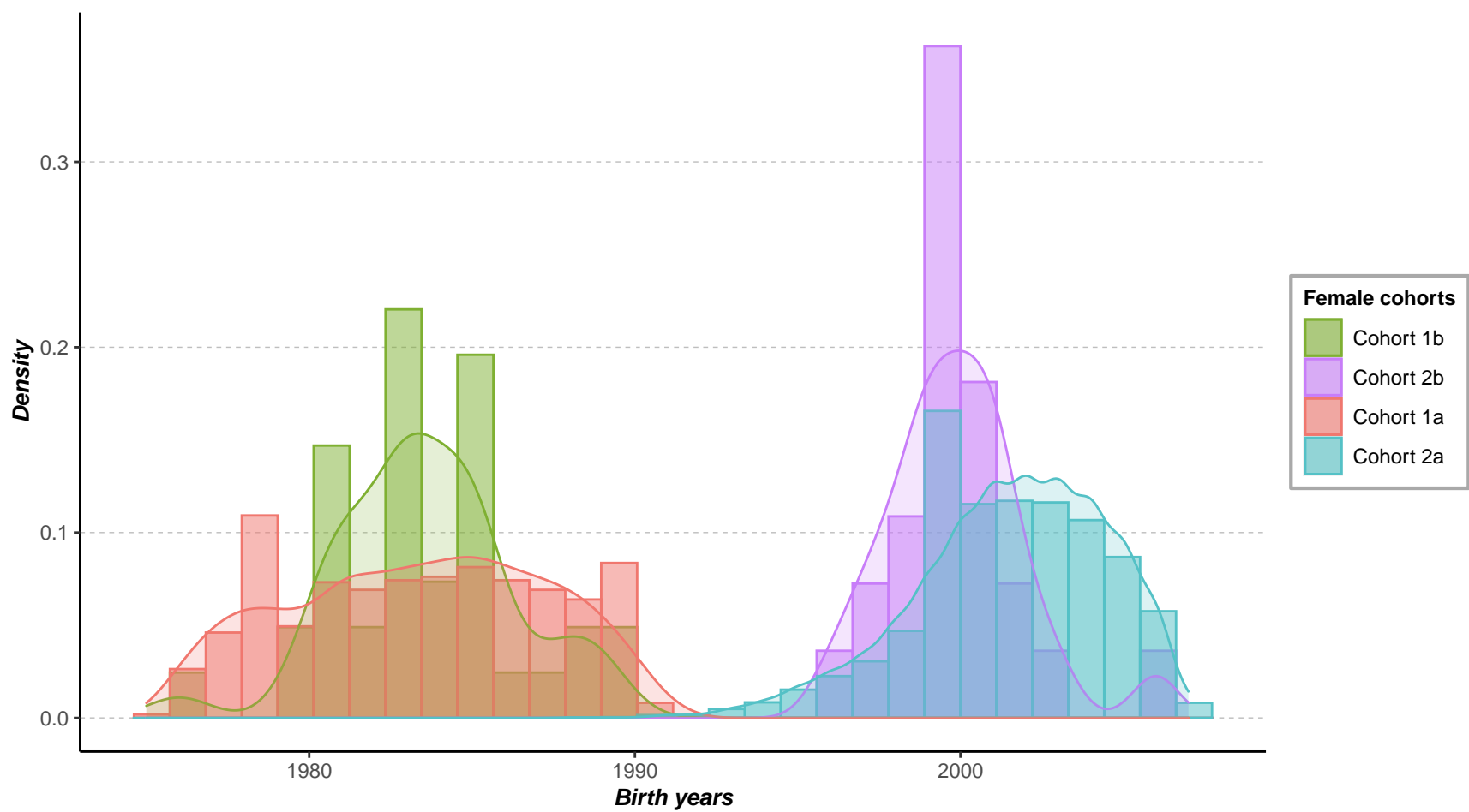

Supplement: Supplementary file 2 — Additional file 2: Figure S2. Distribution of birth years for the four female cohorts. The 2443 cows in Cohort 1a are represented in pink, the 4092 cows in Cohort 2a are represented in blue, the 37 cows in Cohort 1b are represented in green and the 25 cows in Cohort 2b are represented in purple. [file 12711_2023_801_MOESM2_ESM.pdf]

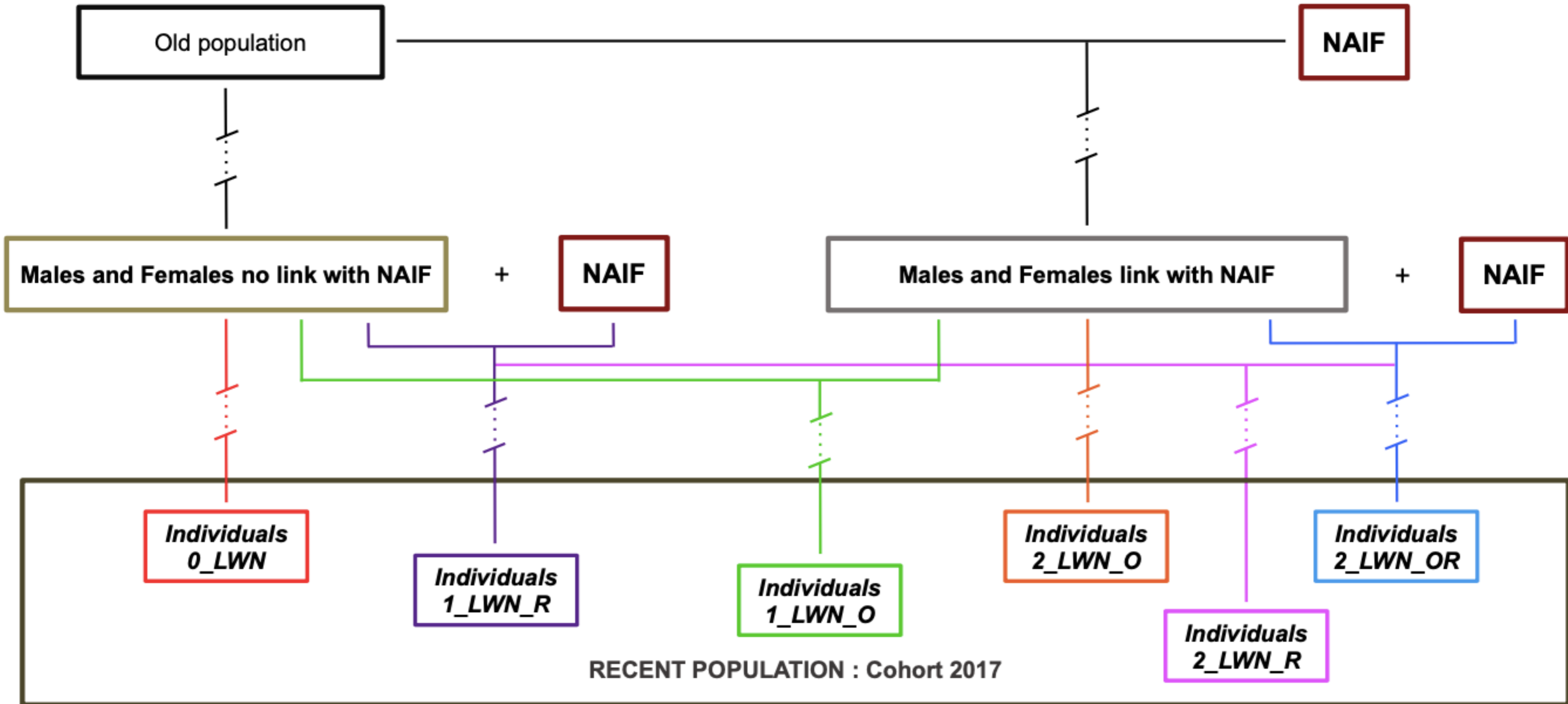

Supplement: Supplementary file 3 — Additional file 3: Figure S3. Classification of different genetical links with the Naif bull for the 2017 cohort. Red: individuals with no genetic family link to Naif (0_LWN); purple: individuals with a recent genetic link with Naif through one of their parents (1_LWN_R); green: individuals with an old link with Naif through one of their parents (1_LWN_O); orange: individuals related to the first use of Naif by both parents (2_LWN_O); magenta: individuals related to the recent use of Naif by both parents (2_LWN_R); blue: individuals with one old and one recent genetic link with Naif (2_LWN_OR). [file 12711_2023_801_MOESM3_ESM.pdf]

*Equivalent number of generations*

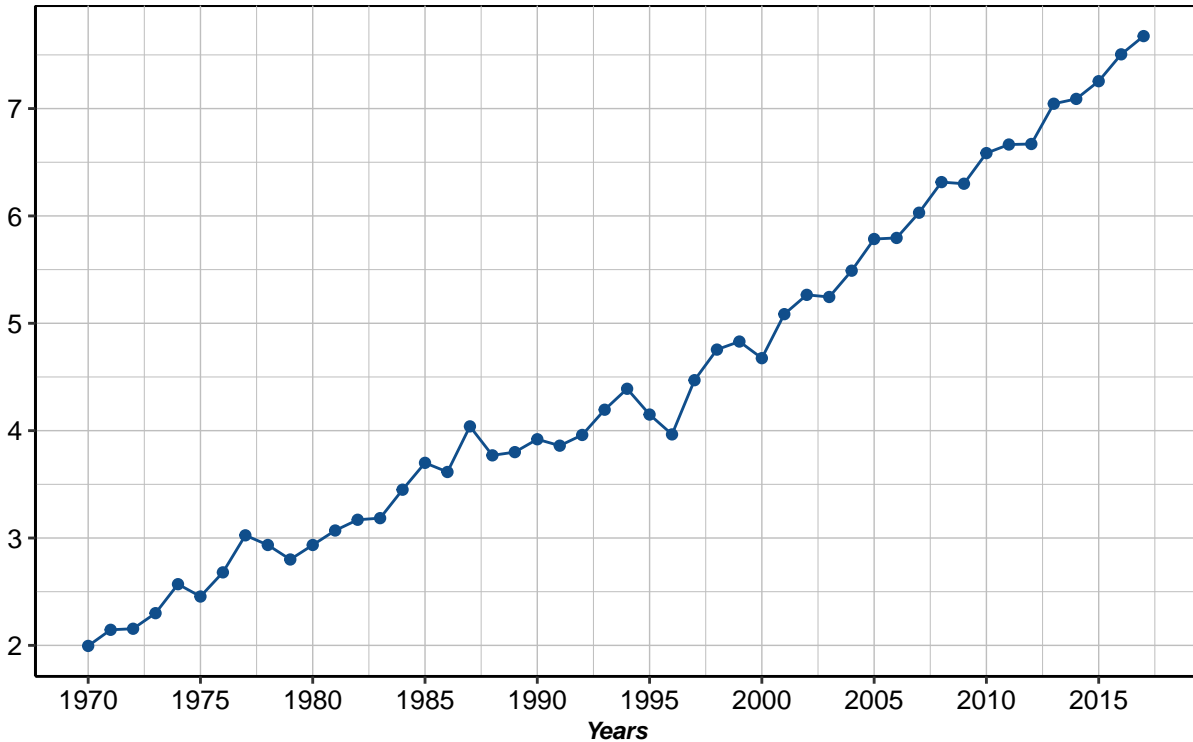

Supplement: Supplementary file 4 — Additional file 4: Figure S4. Annual evolution of the equivalent number of generations from the pedigree data. [file 12711_2023_801_MOESM4_ESM.pdf]

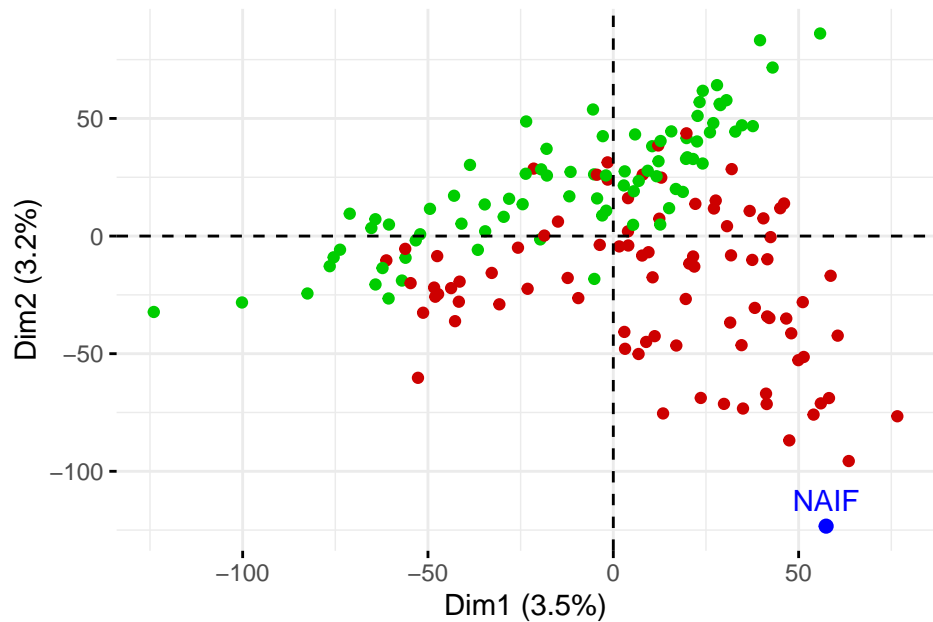

Link with NAIF

- LWN (85 ind)
- no\_LWN (80 ind)

NAIF

NAIF in darkblue

Supplement: Supplementary file 5 — Additional file 5: Figure S5. Principal component analysis of genotyping data for Cohort 2. Green: individuals with no genetic link to Naif (noLWN); and red: individuals with a genetic link to Naif (LWN). Naif is represented by the blue dot. [file 12711_2023_801_MOESM5_ESM.pdf]

*INEL*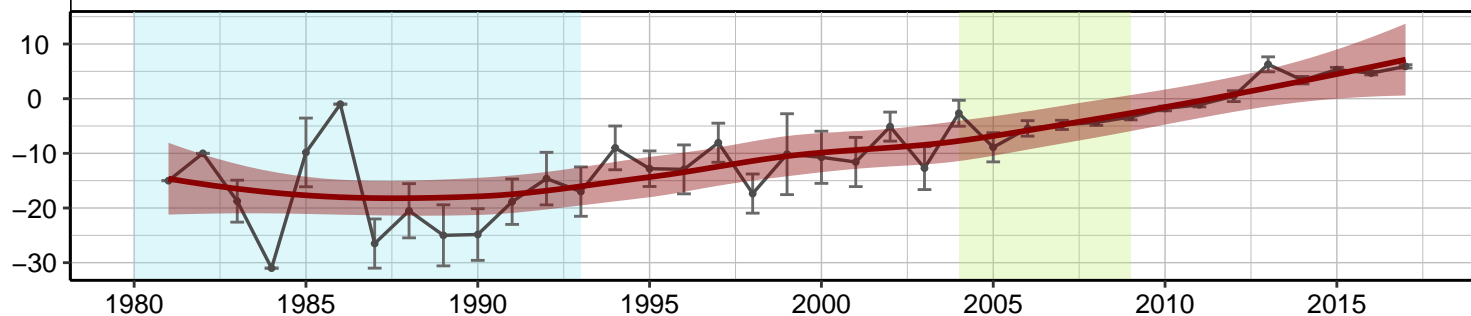*ISU*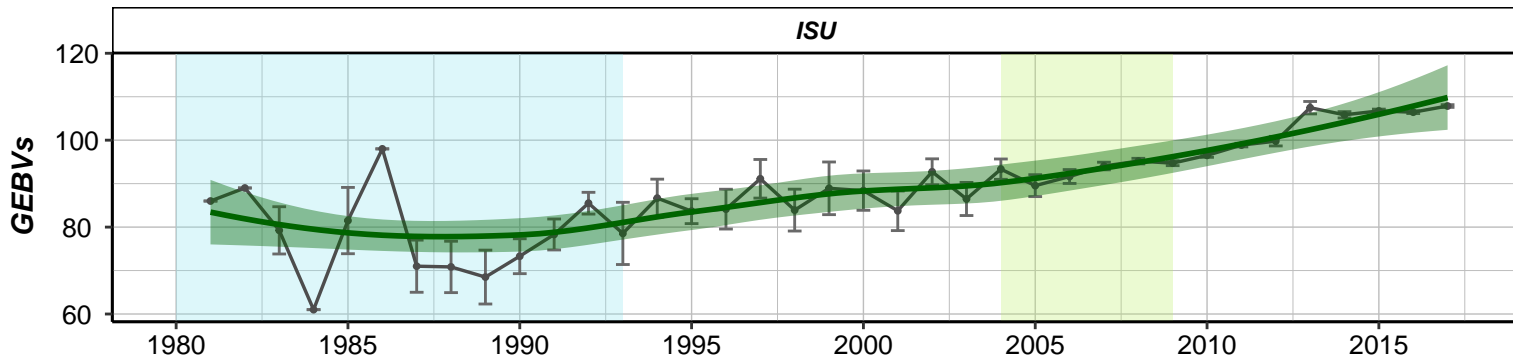*REPRO*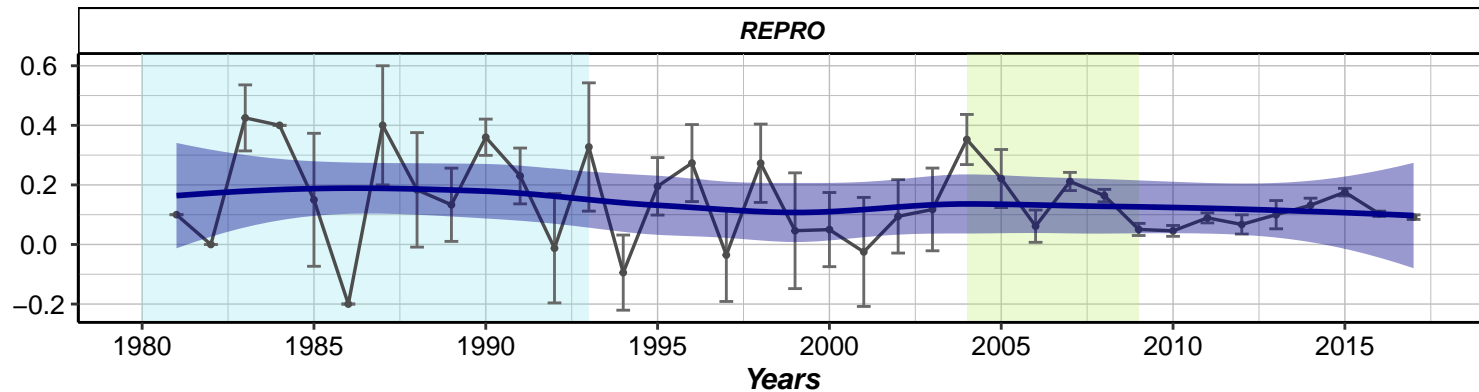

Supplement: Supplementary file 8 — Additional file 8: Figure S6. Evolution of GEBV for the three indices (INEL, ISU, REPRO) in the Abondance breed. GEBV were assessed in 2017 for all genotyped individuals. Error bars correspond to standard errors. [file 12711_2023_801_MOESM8_ESM.pdf]

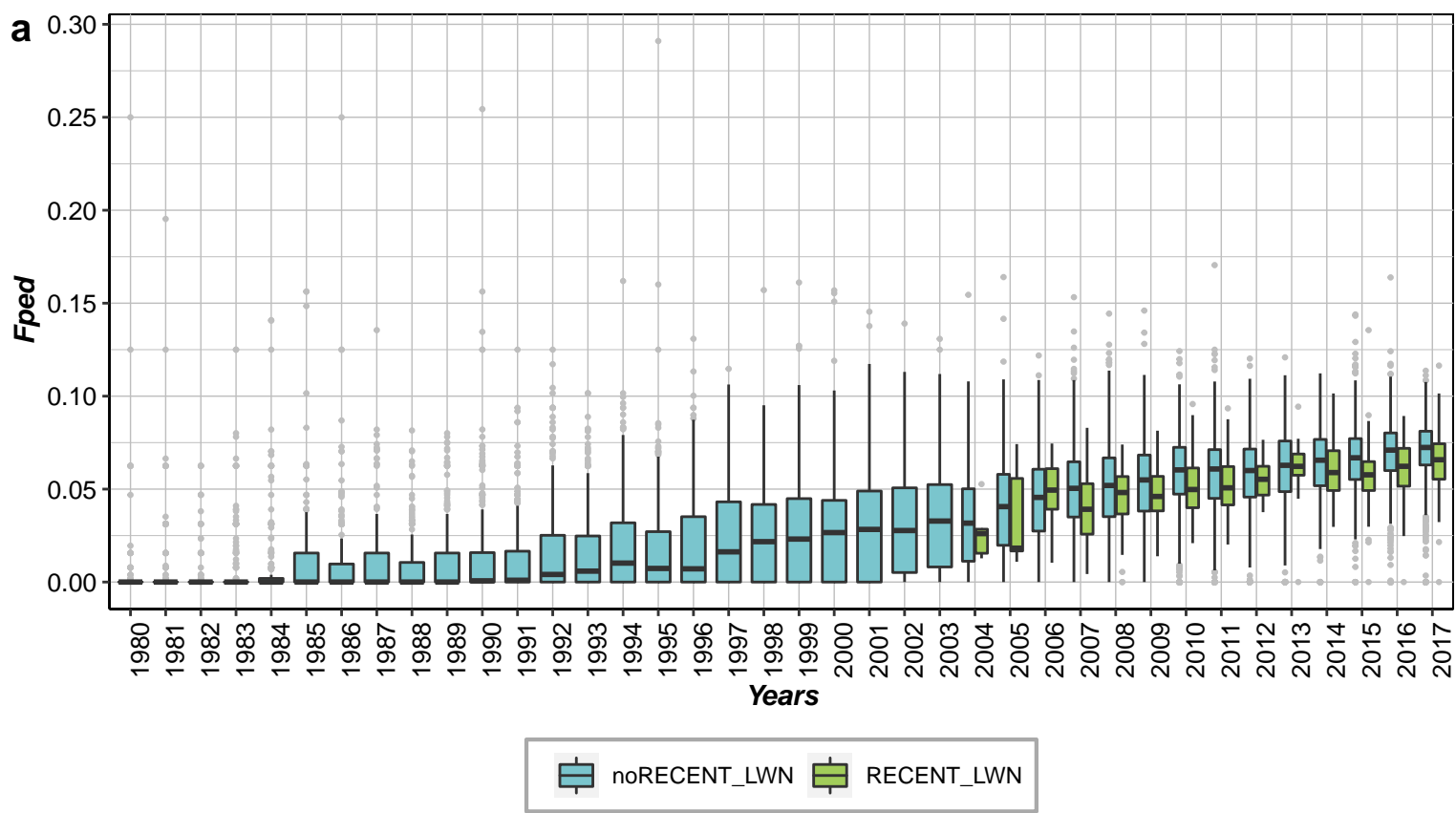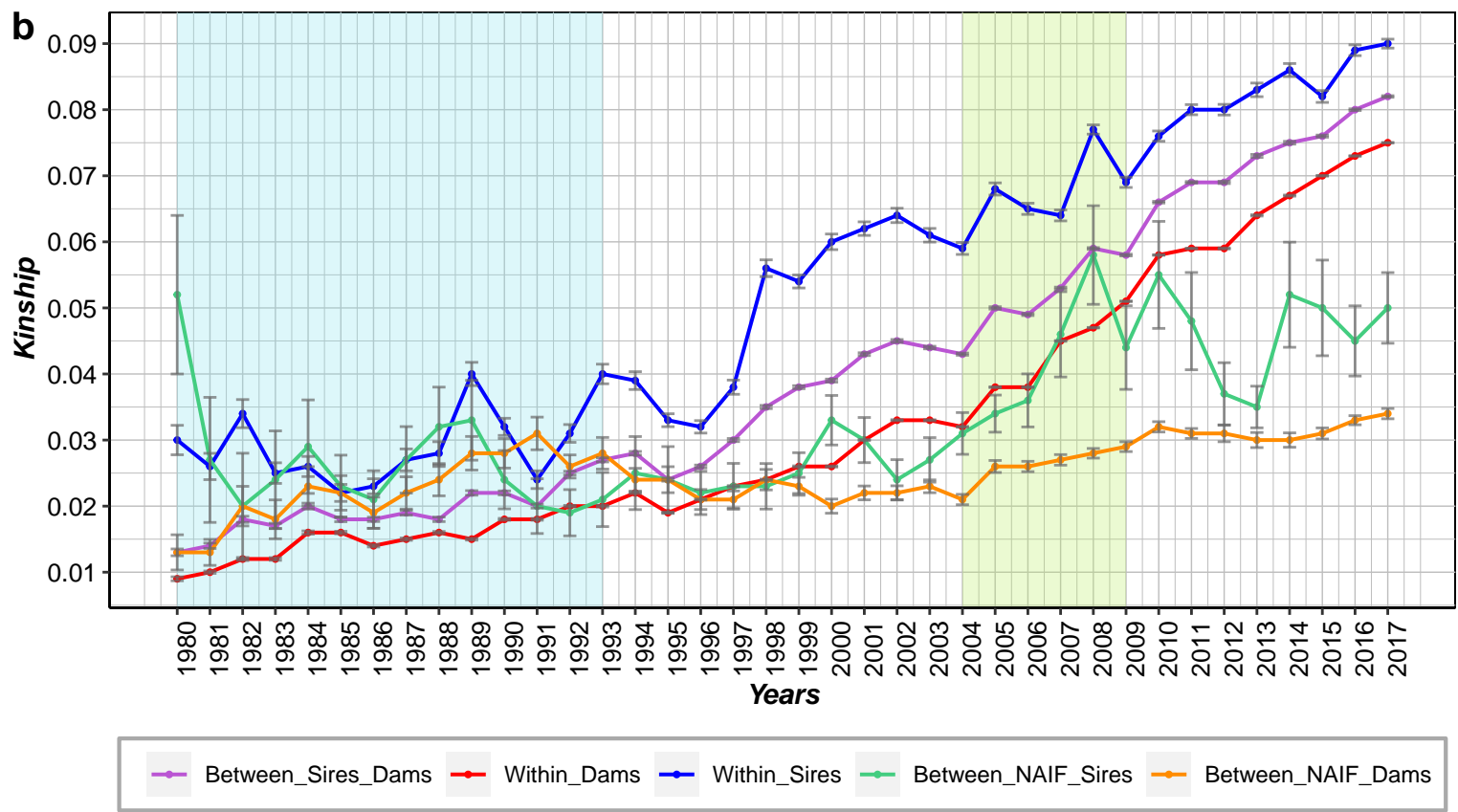

Supplement: Supplementary file 9 — Additional file 9: Figure S7. Annual evolution of average inbreeding (a) and average kinship (b) according to the re-use of Naif frozen semen. Inbreeding and relatedness values were evaluated from pedigree using the MEUW and PAR3 modules, respectively, of the PEDIG software. (a) Individuals resulting from the recent use of Naif are represented in green and individuals not resulting from the recent use of Naif are represented in blue. (b) Kinship within sires is shown in blue, kinship within dams is shown in red, and kinship between sires and dams is in purple. The kinships between Naif and other sires are represented in green. The kinships between Naif and dams are represented in orange. Error bars correspond to standard errors. [file 12711_2023_801_MOESM9_ESM.pdf]

*REPRO*

*Inbreeding – FROH*

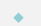

noRECENT\_LWN

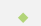

RECENT\_LWN

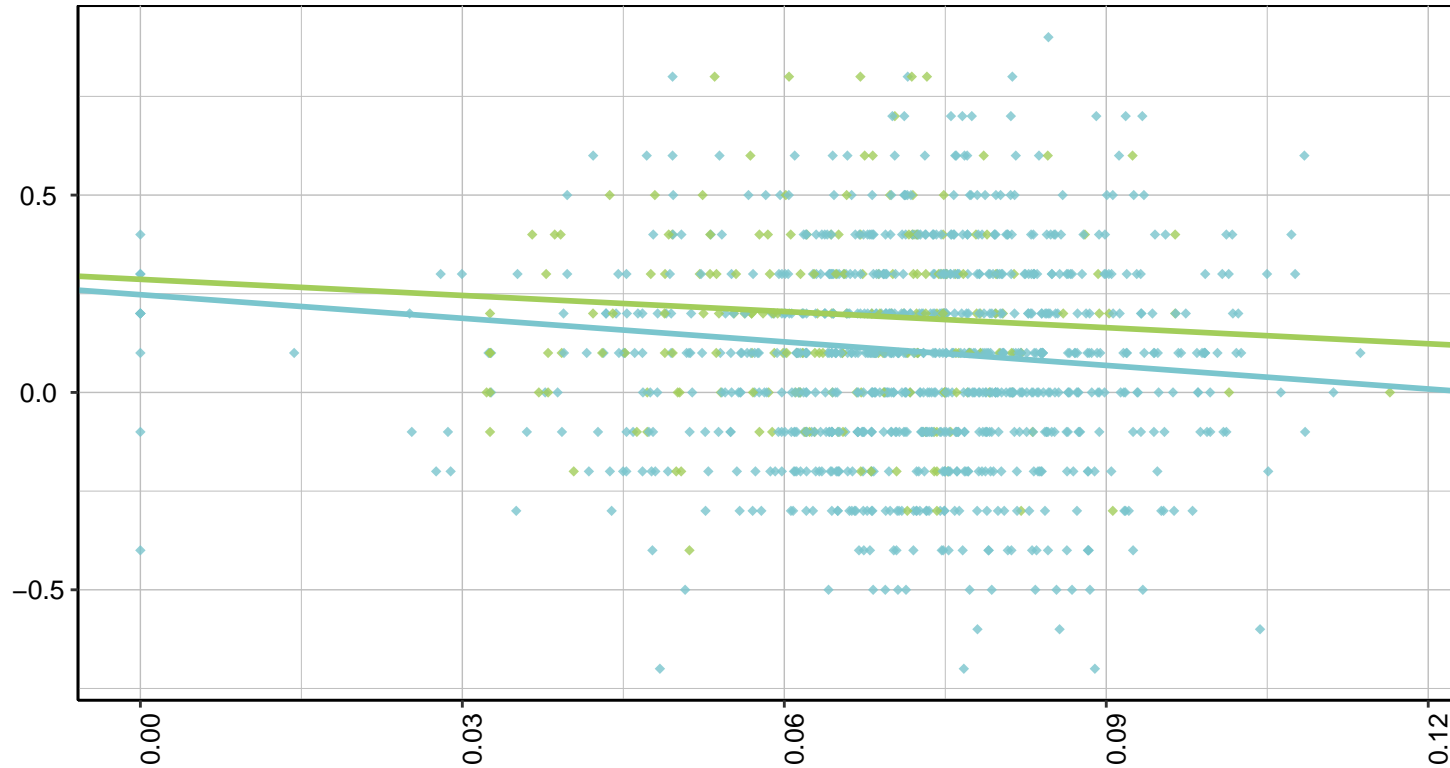

Supplement: Supplementary file 10 — Additional file 10: Figure S8. Inbreeding depression on reproduction index in 2017 for individuals resulting or not from the reintroduction of the Naif bull. Blue: individuals not resulting from the recent use of Naif—Green: individuals resulting from the recent use of Naif. Regression lines were estimated using an ANCOVA model considering reproduction index (REPRO) as the response variable, and the inbreeding level based on ROH (FROH), the link to the recent use of Naif (NoRECENT_LWN if no link and RECENT_LWN if resulting for reintroduction of Naif) and its interaction as explanatory variables. In blue, $REPRO_{noRECENT\_LWN} = 0.25 - 1.99F_{ROH}$REPROnoRECENT_LWN=0.25-1.99FROH. In green, $REPRO_{noRECENT\_LWN} = 0.29 - 1.36F_{ROH}$REPROnoRECENT_LWN=0.29-1.36FROH. The slopes of the regression lines were not significantly different between the two groups (p = 0.63). [file 12711_2023_801_MOESM10_ESM.pdf]
